# Low Hepatitis B vaccination rates among medical students in South Asia: A systematic review and meta-analysis

**DOI:** 10.1371/journal.pone.0320330

**Published:** 2025-03-25

**Authors:** Ramesh Lamichhane, Indra Dev Pathak, Bishnu Deep Pathak, Pritha Adhikari, Sagun Dawadi, Aashika Rai, Pratikshya Ojha, Kripa Maharjan, Kirtan Gautam, Nishan Dhakal, Madhusudan Saha

**Affiliations:** 1 Department of Gastroenterology and Hepatology, Mayo Clinic, Rochester, United States of America; 2 Department of Internal Medicine, Nepalese Army Institute of Health Sciences, College of Medicine, Kathmandu, Nepal; 3 Department of Pediatrics, SUNY Upstate Medical University, New York, United States of America; 4 Department of Gastroenterology and Hepatology, Sylhet Women’s Medical College, Sylhet, Bangladesh; Mount Sinai Hospital, UNITED STATES OF AMERICA

## Abstract

**Background and aims:**

Current and future healthcare professionals, such as medical students, are at risk of contracting Hepatitis B virus infection. Vaccination against Hepatitis B is an effective means of prevention. However, studies have reported variable vaccination rates among medical students from different regions of South Asia. Understanding vaccination rates and barriers can guide effective interventions to protect future doctors. Therefore, we aimed to find out the vaccination rate among medical students in South Asia.

**Methods:**

A comprehensive literature search was conducted across multiple databases (PubMed, PubMed Central, Scopus, Embase, CINAHL, Google Scholar, MEDLINE, and other sources) beginning from inception to July 15, 2024. Observational studies reporting vaccination rates among Bachelor of Medicine and Bachelor of Surgery (M.B.B.S.) students in South Asia were included. Two reviewers independently screened and performed the quality assessment. Pooled vaccination rates were calculated and visualized using a random-effects model in R Studio (Version 2023.12.0). Subgroup analysis was performed based on country and year of publication of the studies.

**Results:**

Fifty studies from the South Asian region, including 12,231 participants, were included in the quantitative analysis. The overall pooled Hepatitis B vaccination rate using the random-effects model was 56% (CI: 49-63%), with significant heterogeneity among the included studies (I^2^ = 98%; *P* = 0). Subgroup analysis based on country revealed a significant regional variation in vaccination rate (59% in Pakistan, 57% in India, 55% in Nepal, and 41% in Bangladesh; *P* < 0.01). Lack of awareness, perceived low risk or necessity, concerns about side effects, logistical challenges, and lack of motivation have been reported as barriers to vaccination.

**Conclusion:**

The overall vaccination rate was relatively low in South Asia, with significant regional variation. Based on the reported barriers, we recommend that relevant authorities focus on vaccination awareness, motivation, cost-effectiveness, logistics management, policy formulation, and monitoring.

## 1. Introduction

Hepatitis B is a widely prevalent global health concern, with approximately 40 million people infected with chronic Hepatitis B in South Asia [1]. Countries within this region, including Afghanistan, Bangladesh, Bhutan, India, Maldives, Nepal, Pakistan, and Sri Lanka, fall into intermediate to low endemic zones, with a prevalence of less than 7% [1]. Healthcare workers and medical students are found to have higher risk due to frequent exposure in clinical settings, with healthcare workers being 2-10 times more likely to acquire Hepatitis B infection compared to general public [2]. The transmission method could be through direct contact with blood or needle stick injuries [3]. Presumably, transition from the preclinical to clinical years is a vulnerable period for medical students because of their added ward duties and interventional procedures.

Vaccination against Hepatitis B is considered a key measure for its prevention; however, studies have reported variable vaccination rates among medical students [4]. In addition, students’ knowledge of Hepatitis B infection and its prevention is reportedly suboptimal [5]. A low vaccination rate and a lack of awareness increase the risk of transmission. Therefore, we aimed to review overall vaccination rates against Hepatitis B among M.B.B.S. students in South Asia. Furthermore, this review highlights the possible existing barriers and proposes a list of strategies for improving vaccination rates in the South Asia region.

## 2. Materials and methods

We used the Preferred Reporting Items for Systematic Review and Meta-Analysis (PRISMA) guidelines (S1 Table) [6]. The study protocol was registered beforehand in the International Prospective Register of Systematic Reviews (PROSPERO, CRD42024569123) [7].

### 2.1. Criteria for inclusion of studies

#### 2.1.1. Types of studies.

Observational studies reporting vaccination rates among medical students were included in this review. Case reports, case series, comments, and systematic reviews were excluded.

#### 2.1.2. Participants.

We included M.B.B.S. students enrolled in medical schools across countries in South Asia irrespective of the academic years they are enrolled in.

#### 2.1.3. Outcomes.

The primary outcome of interest was the overall pooled rate of Hepatitis B vaccination among medical students in South Asia. Additional outcomes included pooled vaccination rates among preclinical (first and second year) and clinical (third year onwards) students as well as rates by individual South Asian countries.

### 2.2. Search methods for identification of studies

A Comprehensive literature search was performed in PubMed, PubMed Central, Scopus, Embase, MEDLINE, CINAHL, and Google Scholar for studies published from inception to July 15, 2024. Additionally, we used a citation search to find any relevant studies that might have been missed in the above database.

#### 2.2.1. Electronic searches.

Details of the search strategy are documented in S1 File.

### 2.3. Data analysis

#### 2.3.1 Selection, data extraction, and management of studies.

The relevant studies from the database search were imported into the Covidence systematic review software for screening and extraction [8]. After the removal of potential duplicates, the title/abstract and full-text reviews of the studies were independently performed by two reviewers (IDP and AR), and the conflicts (if any) were resolved by the third reviewer (RL). The inclusion criteria were as follows:

i Studies conducted in South Asian countries (Nepal, India, Pakistan, Bangladesh, Sri Lanka, Bhutan, Maldives, Afghanistan) with reported Hepatitis B vaccination rates among medical students.
ii Cross-sectional, cohort, editorials (including description of cross-sectional study), and other observational studies that have reported vaccination rates.

The exclusion criteria were as follows:

i Studies not including South Asian medical student population.
ii Studies without reported Hepatitis B vaccination rates.
iii Case reports, case series, review articles, meta-analysis, and randomized clinical trials.

After full-text review, relevant data from each included study were extracted independently into Microsoft Excel spreadsheets by two reviewers (IDP and KG). Disagreements on the extracted data were resolved by the third reviewer (ND).

#### 2.3.2 Assessment of risk of bias in included studies.

The evaluation of quality of the included studies was carried out independently by two reviewers (BDP and SP). The critical appraisal tool from the Joanna Briggs Institute (JBI) for prevalence studies was used to assess the risk of bias [9]. The table outlining the risk of bias is provided in S2 Table.

#### 2.3.3 Assessment of heterogeneity.

Heterogeneity in the included studies was determined using the I^2^ test outlined in the Cochrane Handbook for Systematic Reviews of Interventions [10]. A heterogeneity above 50% was considered significant, and a DerSimonian-Laird method was used under random-effects model to calculate pooled effect size and other analyses.

#### 2.3.4 Data synthesis.

The extracted data were analyzed using Metapackage in RStudio (Version 2023.12.0) [11]. Continuous variables were presented as mean/standard deviation or median/range, while categorical variables were presented as frequency and proportion/percentage. The mean pooled prevalence of Hepatitis B vaccination rates was calculated and depicted as the pooled mean prevalence with 95% CI using a random-effects model. The publication bias was assessed using Egger’s regression asymmetry test and was visualized using a funnel plot. Likewise, sensitivity analysis was performed using leave-one-out method, excluding one study at a time.

## 3. Results

### 3.1 Study selection

**Fig 1:**
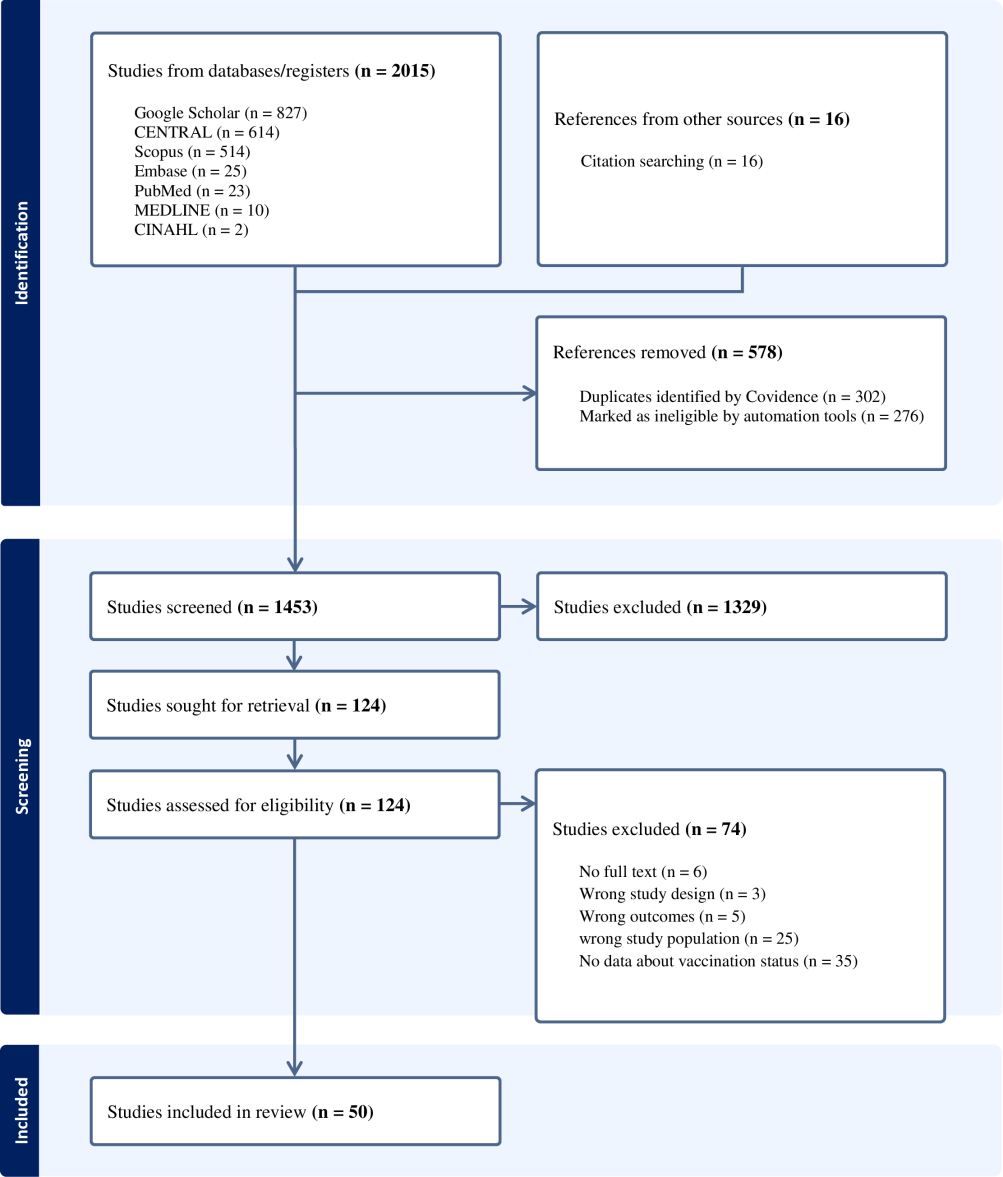
PRISMA flow diagram.

A total of 2,015 studies were imported into Covidence software from database searches on PubMed, PubMed Central, Scopus, Embase, CINAHL, Google Scholar, and MEDLINE. After removing duplicate records, 1,453 studies were screened by their titles and abstracts. Additionally, 16 studies obtained from direct citation searching were also imported. Of these, 124 studies were eligible for full-text screening. Among these, 74 studies were excluded because they did not meet the eligibility criteria. Finally, 50 were included in the quantitative data analysis. The details of the study selection are shown in **Fig 1**.

### 3.2 Study characteristics

The total sample size across all the studies (n = 50) was 12,231, ranging from 50 to 1,509. We included studies conducted in South Asian countries, with eligible studies from India, Pakistan, Nepal, and Bangladesh. The studies from other South Asian countries did not meet the inclusion criteria. The characteristics of the individual studies are summarized in **Table 1**.

**Table 1:** Characteristics of the included studies.

Author^a^	Publication Year	Country	Age (years)	Sex Distribution (%)	Sample Size (n)	Vaccinated Participants, n (%)
Acchammachary et al. [12]	2017	India	–	Male-56% Female-44%	118	57 (48.30)
Pattanshetty et al. [13]	2010	India	–		570	452 (79.30)
Pavani et al. [14]	2015	India	18 to 25	–	96	35 (36.46)
Gujjarlapudi et al. [15]	2013	India	21 ± 1.3	Male-45% Female-55%	200	85 (42.50)
Tariq et al. [16]	2017	Pakistan	–	Male-48% Female-52%	167	120 (71.86)
Agarwal et al. [17]	2018	India	–	–	200	145 (72.50)
Baig et al. [18]	2015	India	26 ± 9	Male-67% Female-33%	354	290 (81.92)
Rathi et al. [19]	2018	India	17 to 25	Male-70% Female-30%	161	13 (8.07)
Suganthi et al. [20]	2014	India	18 to 20	Male-53% Female-47%	120	21 (17.50)
Mishra et al. [21]	2019	India		–	100	81 (81.00)
Pulluri et all. [22]	2017	India	18	Male-41% Female-59%	147	70 (47.62)
Wadekar et al. [23]	2019	India	–	–	136	133 (97.79)
Diwan et al. [24]	2019	Pakistan	–	–	86	44 (51.16)
Khan et al. [25]	2013	Pakistan	–	–	1509	825 (54.67)
Shahbaz et al. [26]	2014	Pakistan	–	–	280	232 (82.86)
Dawar et al. [27]	2019	India	–	–	106	87 (82.08)
Giri et al. [28]	2016	India	18 to 26	Male-54% Female-46%	110	57 (51.82)
Nowreen et al. [29]	2019	India	20 ± 3	Male-46% Female-54%	127	59 (46.46)
Batra et al. [30]	2015	India	–	–	173	108 (62.43)
Shahid et al. [31]	2019	Pakistan	17 to 26	–	300	150 (50.00)
Biradar et al. [32]	2015	India	–	–	72	43 (59.72)
Nasir et al. [33]	2000	Pakistan	–	Male-58% Female-42%	327	138 (42.20)
Asif et al. [34]	20011	Pakistan	20 to 30	Males-57% Females-43%	375	186 (49.60)
Butt et al. [35]	2015	Pakistan	–	Male-44% Female-56%	269	89 (33.09)
Thote et al. [36]	2023	India	19 to 21	Male-62% Female-38%	437	56 (12.81)
Shrestha et al. [4]	2020	Nepal	20 ± 2	Male-68% Female-32%	181	67 (37.02)
Bhattarai et al. [37]	2014	Nepal	–	–	340	290 (85.29)
Sajid et al. [38]	2018	Pakistan	–	Male-44% Female-56%	467	318 (68.09)
Pratap et al. [39]	2024	India	–	Male-44% Female-56%	317	142 (44.79)
Riaz et al. [40]	2017	Pakistan	20 to 26	Male-45% Female-57%	126	93 (73.81)
Khan et al. [41]	2018	Pakistan.	17 to 29	Male-50% Female-50%	150	90 (60.00)
Sayeed et al. [42]	2007	Bangladesh	18 to 19	Male-54% Female-46%	167	62 (37.13)
Paul et al. [43]	2015	India	18	–	144	143 (99.31)
Malik et al. [44]	2017	Pakistan	17 to 26	Male-43% Female-57%	160	47 (29.38)
Bhattarai et al. [45]	2020	Nepal	24 ± 3	–	204	96 (47.06)
Hussain et al. [46]	2016	India	19 to 25	Male-33% Female-67%	341	91 (26.69)
Dahal et al. [47]	2024	Nepal	23 ± 2	Male-67% Femal-33%	206	96 (46.60)
Raza et al. [48]	2008	Pakistan	21 ± 3	Male-37% Female-63%	900	726 (80.67)
Gautam et al. [49]	2022	India	–	–	150	24 (16.00)
Sachidananda et al. [50]	2022	India	–	–	403	211 (52.36)
Chhabra et al. [51]	2019	India	21 ± 6	Male-67% Female-33%	180	149 (82.78)
Jayakiruthiga et al. [52]	2018	India	18 to 21	Male-48% Female-52%	200	64 (32.00)
Avachat et al. [53]	2017	India	–	–	140	68 (48.57)
Gupta et al. [54]	2017	India	21 to 24	Male-47% Female-53%	140	130 (92.86)
Singh et al. [55]	2011	India	21 ± 2	–	138	94 (68.12)
Daud et al. [56]	2007	Pakistan	–	–	50	33 (66.00)
Ahmed et al. [57]	2009	Bangladesh	19 ± 1	Male-53% Female 47%	100	33 (33.00)
Pantha et al. [58]	2011	Bangladesh	–	Male-51% Female-49%	125	64 (51.20)
Srinivas et al. [59]	2021	India	–	–	145	116 (80.00)
Akhter et al. [60]	2016	Bangladesh	22 ± 3	Male-36% Female-64%	217	90 (41.47)

Age is in years, with median/range or mean ±  standard deviation.

^a^All studies were cross-sectional in design.

### 3.3 Quality assessment

The quality of the included studies was assessed by using JBI checklist for prevalence studies [9]. The overall appraisal was good, and all the studies were included in the final analysis. The detailed assessment and appraisal have been presented in S2 Table. A major concern was that some of the studies[13–15,18–20,22–25,29–31,38–42,46,54,57,60,61] lacked proper explanation of their sampling methods and sample size which could potentially bring bias and can affect the generalizability of our findings.

#### 3.4 Prevalence of rate of hepatitis b vaccination.

**Fig 2:**
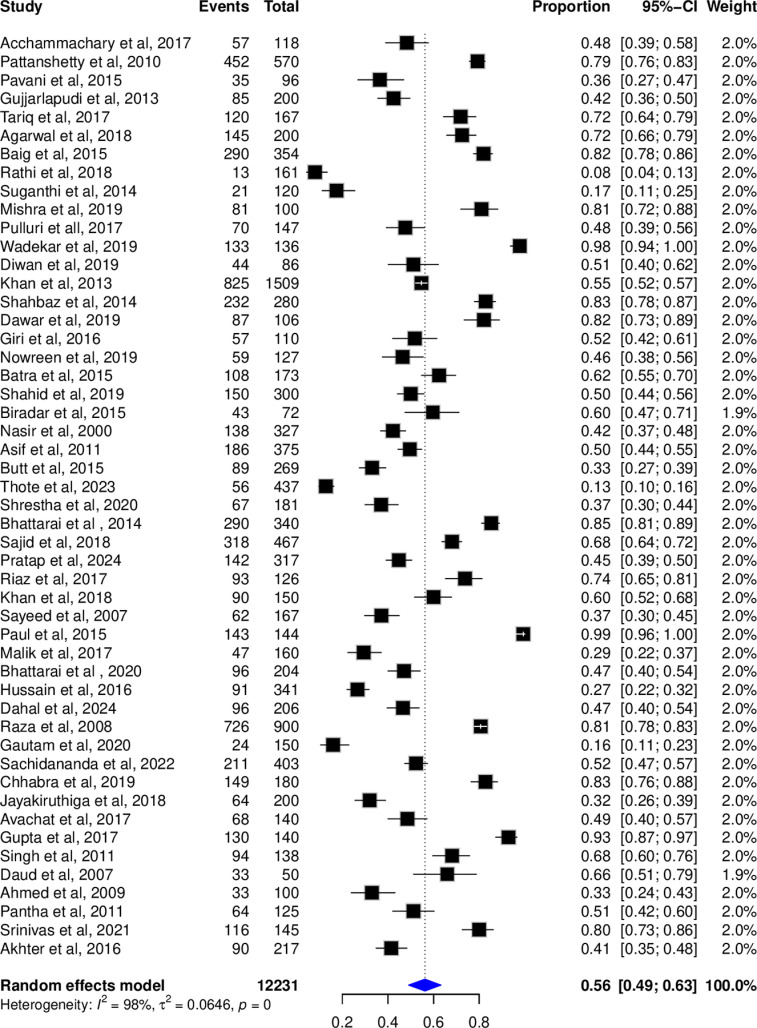
Forest plot representing the pooled vaccination rates across studies.

The pooled Hepatitis B vaccination rate using random-effects model was 56% (95% CI; 49-63%), with significant heterogeneity across the included studies (I^2^ = 98%; *P* = 0) (**Fig 2**).

#### 3.5 Barriers to vaccination.

The barriers to vaccination in studies can be grouped into several categories: **Lack of Awareness and Knowledge**, including issues such as unawareness of vaccination status, inadequate awareness of benefits, and not knowing where to get the vaccine; **Perceived Low Risk or Necessity**, with many students not feeling the need for vaccination, showing casual behavior, or not considering it necessary; **Concerns About Safety and Side Effects**, such as fears of side effects, needles, or adverse reactions, along with a lack of belief in vaccination; **Logistical Challenges**, including high vaccine costs, lack of easy access, distant vaccination centers, lack of vaccination programs, and lack of reminders; and **Motivational Factors**, where issues such as motivational weakness, laziness, and forgetfulness prevent individuals from getting vaccinated [4,16,19,27,32,35,37,45,55,58,60,62,63].

### 3.6 Subgroupanalysis

#### 3.6.1 Subgroup analysis by study region.

**Fig 3:**
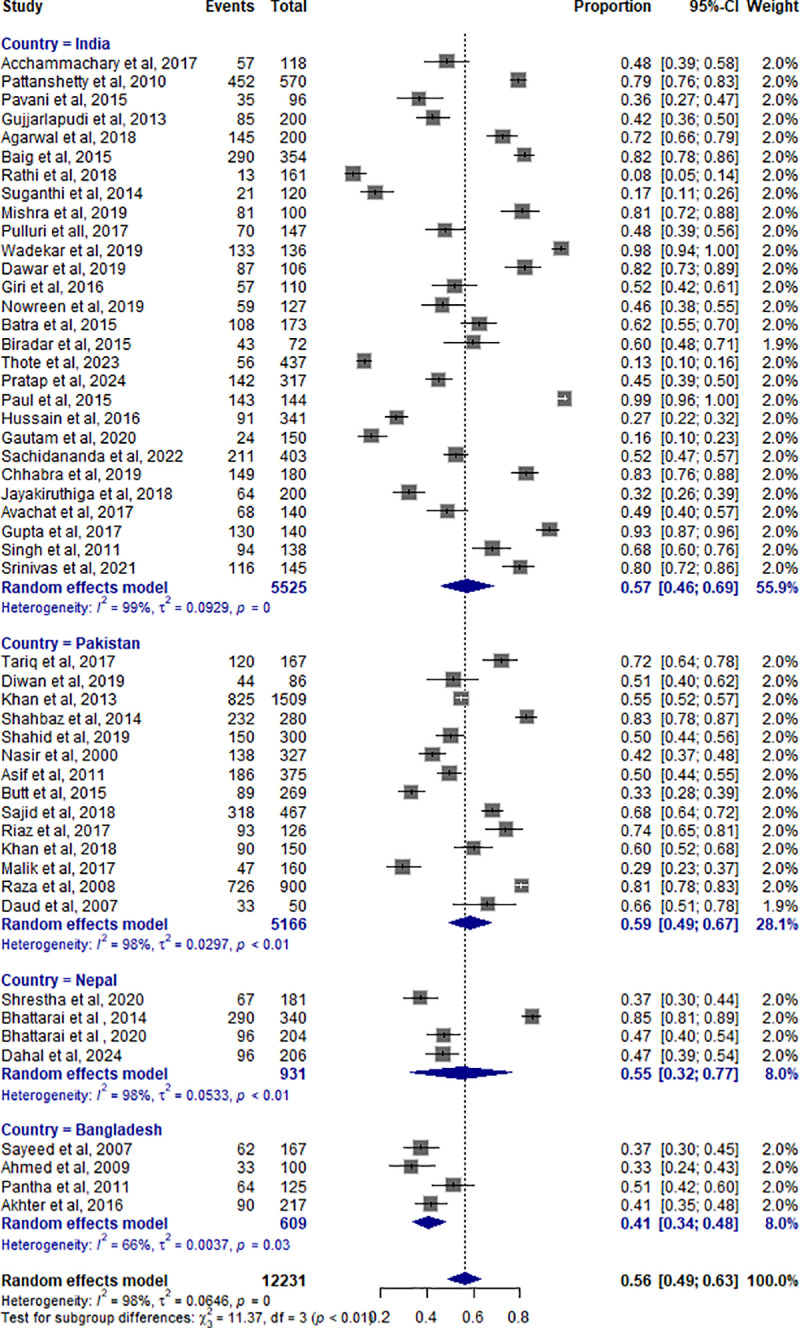
Forest plot of pooled vaccination rates in different South Asian countries.

The subgroup analysis was conducted based on country. The pooled prevalence of vaccination rate was 0.59 (95% CI: 0.49-0.67) for Pakistan, 0.57 (95% CI: 0.46–0.69) for India, 0.55 (95% CI: 0.32-0.77) for Nepal, and 0.41 (95% CI: 0.34–0.48) for Bangladesh. Heterogeneity (I²) was 66-98% across the groups. There was a significant difference among the countries (χ² =  11.37, df =  3, *P* < 0.01), indicating a regional variation (**Fig 3**).

#### 3.6.2 Subgroup analysis by study population.

**Fig 4:**
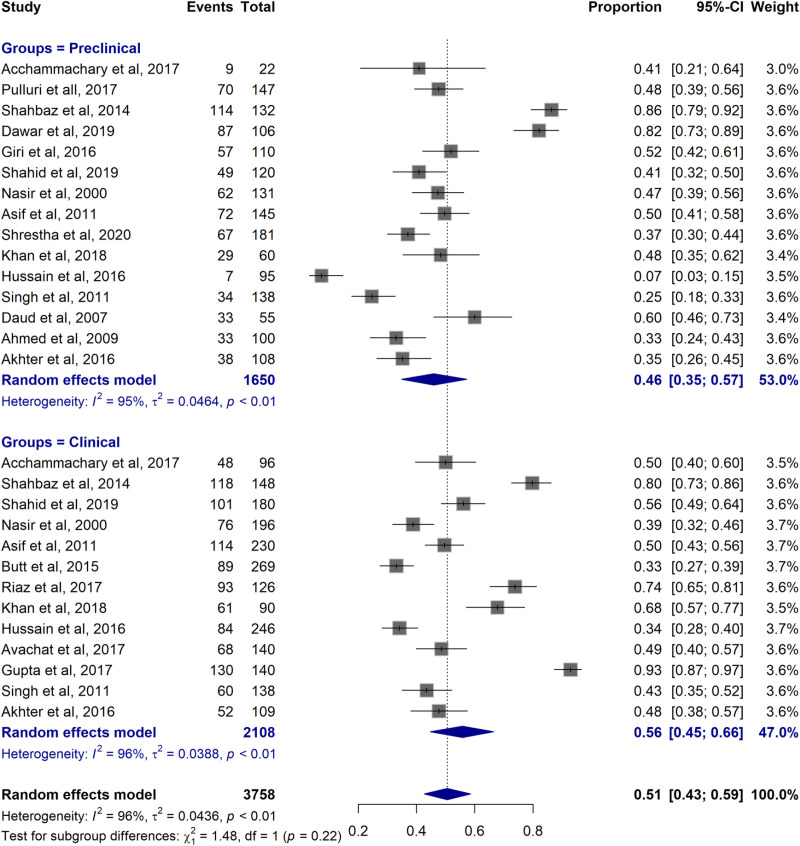
Forest plot of pooled vaccination rates in preclinical and clinical subgroups.

The subgroup analysis compared pooled prevalence of vaccination rates between preclinical and clinical students. The pooled prevalence of Hepatitis B vaccination among preclinical group was 0.46 (95% CI: 0.35–0.57) and among clinical students was 0.56 (95% CI: 0.45–0.66) with a significant heterogeneity (I² ≥ 95%, *P* < 0.01) across the studies. However, no significant difference was observed between the subgroups (χ² =  1.48, df =  1, *P* = 0.22) (**Fig 4**).

#### 3.6.3 Subgroup analysis by study year.

**Fig 5:**
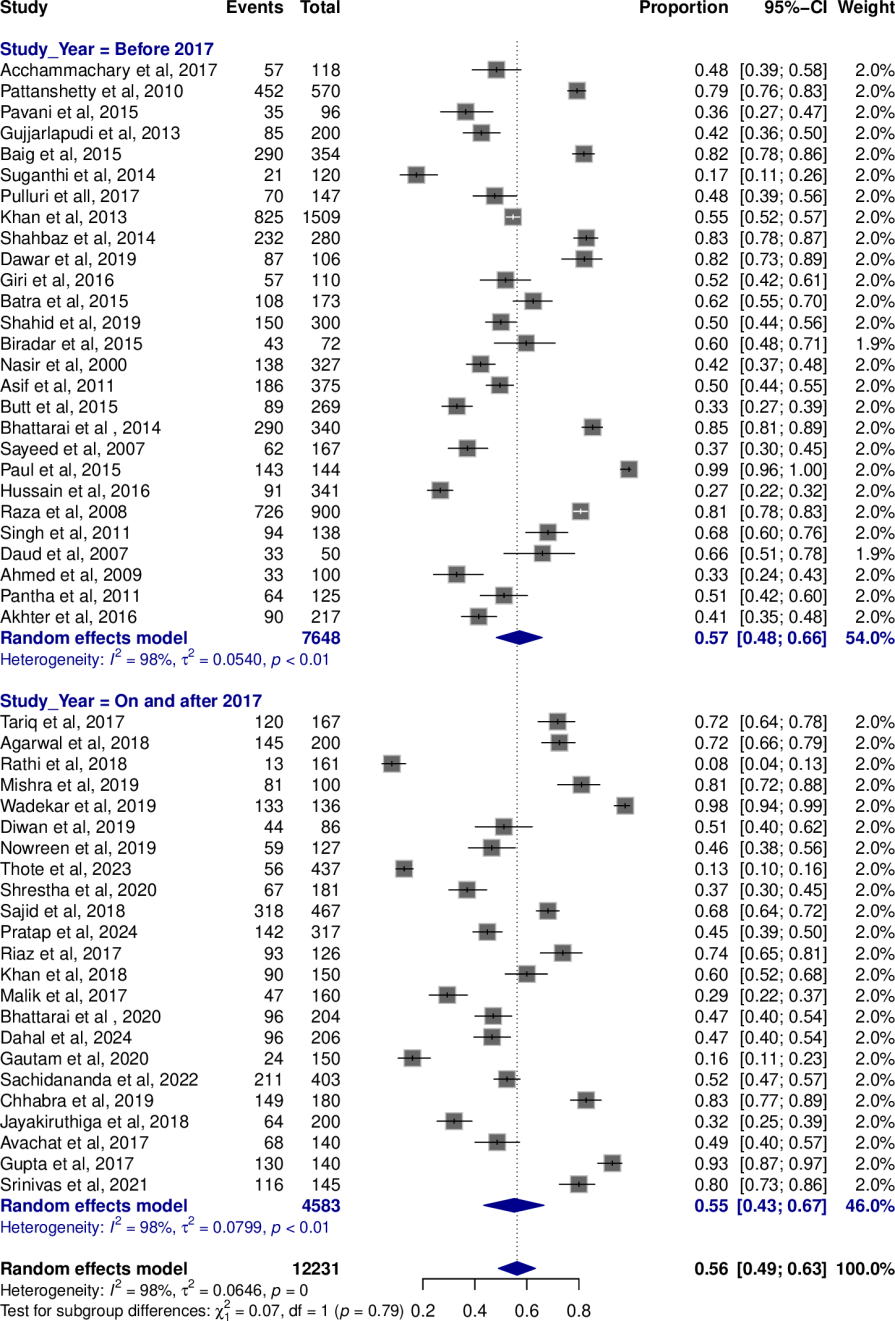
Forest plot of pooled vaccination rate in studies conducted before 2017 and on or after 2017.

During this study, we observed almost similar number of the studies conducted before and after the year 2017, due to which we performed analysis between these two groups to see any pertinent differences in vaccination rate. The studies before 2017 had a pooled vaccination rate of 0.57 (95% CI: 0.48–0.66) with high heterogeneity (I² =  98%, τ² =  0.05, *P* < 0.01). Similarly, the studies from 2017 onwards showed a pooled rate of 0.55 (95% CI: 0.43–0.67) with high heterogeneity (I² =  98%, τ² =  0.07, *P* < 0.01). However, there was no significant difference between these subgroups (χ² =  0.07, df =  1, *P* = 0.79) (**Fig 5**).

#### 3.7 Publication bias and sensitivity analysis.

An Egger’s regression asymmetry test assessed publication bias, showing no significant bias (p =  0.54) (S1 Fig). A funnel plot visualized bias across the 50 studies in the meta-analysis (S2 Fig). The test result (t =  -0.61, df =  48) and bias estimate (-1.88, SE =  3.08) indicated no significant small study effects on the overall findings. Despite residual heterogeneity (τ² =  58.25), the symmetrical funnel plot suggested that publication bias did not likely affect the meta-analysis results. Likewise, the sensitivity analysis was conducted by excluding individual studies (S3 Fig). There was no significant change in the obtained results in any outcome.

## 4. Discussion

In this systematic review, we reviewed 50 different studies incorporating 12,231 participants from four South Asian countries: Nepal, India, Pakistan, and Bangladesh. The overall Hepatitis B vaccination rate among medical students was 56%, ranging from 49% in Bangladesh to 59% in Pakistan. Likewise, vaccination rates among preclinical and clinical students were 47% and 56%, respectively.

All the studies included in this review were conducted over a long period of two and a half decades (1998 to 2024 AD), with an approximately equivalent number of studies being carried out before and 2017 onwards. We found no significant difference in vaccination rates after 2017 compared to that before it (57% vs. 55%, *P* = 0.79). This most likely indicates that priorities over vaccination and prevention against Hepatitis B have not significantly changed over time in South Asia. This finding likely depicts the lack of focus on this matter at both the governmental and local institutional levels.

Although the vaccination rate in our review was numerically higher among clinical (56%) students compared to preclinical ones (47%), there was no significant difference among them, which indicates poor evaluation and preparation during the transition phase of medical education. The clinical phase includes posting in inpatient, outpatient, and emergency departments, where the students are likely to be exposed to patients and their body fluids [3]. Moreover, involvement in invasive procedures and sustaining needle-stick injuries during the initial learning phase further increases the risk of contracting infectious diseases such as Hepatitis B. Therefore, there is a need for proper identification and vaccination of preclinical students before starting their clinical rotations in South Asian countries.

This systematic review showed significant regional variation in Hepatitis B vaccination in South Asia, with pooled vaccination rates of 59%, 57%, 55%, and 41% in Pakistan, India, Nepal, and Bangladesh, respectively. This is a very low value compared with developed countries, which are far more advanced in terms of vaccination campaigns and strategies. A US based study conducted in the 1990s depicted that more than 90% of students were immune to Hepatitis B in a medical institution [64]. A similar study by Yanase et al. from Japan showed that 84% of healthcare personnel in a tertiary medical center reported being vaccinated against Hepatitis B [65].

In our study, it appears that Pakistan has the highest vaccination status, followed by India, Nepal, and subsequently Bangladesh being at the bottom of the list. In this context, most studies from Bangladesh reported lack of awareness/education as a major cause of non-vaccination, while knowledge regarding Hepatitis B seems to be relatively better among students of a few medical colleges in Pakistan, as reported by Malik et al. [44] and Shahbaz et al. [26]. However, this cannot be accurately speculated as a definite reason for the differences in vaccination rates among South Asian countries. Although there are several causes of non-vaccination, lack of awareness has been cited as a common reason in most of the included studies, rendering it a target area of intervention.

Our systematic review has a few limitations that could potentially affect its findings. First, studies from only four South Asian countries were included because no relevant studies on Hepatitis B vaccination status were found in Bhutan, Sri Lanka, Afghanistan, or Maldives. This could possibly limit the external validity of our study. Second, all included studies were of observational type with cross-sectional study designs, and a few of them lacked proper sampling methods and adequate sample size, which may limit the generalizability of our findings. Likewise, various types of data collection tools, including self-report questionnaires, have been used across different studies, which may have introduced reporting and recall bias. Moreover, significant heterogeneity has been consistently found among these studies, which may have biased our results. To overcome these limitations, we conducted a sensitivity analysis that showed no significant deviation in our results, and there was no publication bias across the included studies as well.

Nevertheless, this systematic review is the first of its kind to reflect the Hepatitis B vaccination status among medical students in South Asia, which largely includes developing nations. The overall vaccination rate was low, despite immunization being a high-priority national public health program in these countries. We have suggested some important strategies for enhancing the Hepatitis B vaccination rate among healthcare students who pose a significant risk of contracting this disease during their educational process. A combined effort from the central, regional, and local institutional levels is needed to address this issue effectively and efficiently.

### Recommendation to increase vaccination rates

**Fig 6:**
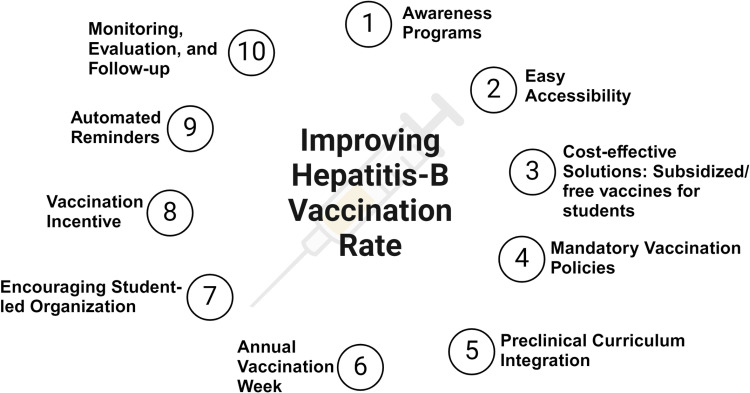
Strategies to increase Hepatitis B vaccination rate.

Based on the assessment of reasons for non-vaccination, we suggest the following ten strategies that aim to increase the vaccination rate among the medical students (**Fig 6**).

1) Awareness programs should be conducted among medical students to improve knowledge regarding Hepatitis B transmission, prevention, and the importance of vaccination, preferably via interactive discussion/workshops. Other examples include distributing pamphlets, printed posters, and through social media posts. Throughout the process, medical students and their peers should be engaged in leadership roles to gain wider attention. To support our statement, evidence suggests structured education program focused on Hepatitis B vaccination have improved students’ knowledge and behavior [66].
2) Easy accessibility: Amidst busy routines, providing vaccination via temporary mobile sites near campuses and student hostels and offering flexible schedules will further enhance vaccination.
3) Cost-effective solutions: To reduce financial burden on students, subsidized/free vaccines should be provided through student insurance, educational institution funds, or partnering with public health agencies.
4) Mandatory vaccination policy: Educational institutions should implement mandatory vaccination policy before starting medical college or transition to clinical training. Policy should allow authorities to implement restrictions to clinical rotations, whenever necessary to enforce timely vaccination.
5) Curriculum integration: Arguably, preclinical to clinical year is the most important transition when vaccination should be ensured because of clinical duties and infected patient exposure. Mandatory modules in the curriculum that comprehensively provide knowledge and address misunderstandings are critical for advocating vaccination.
6) Annual vaccination week: July 28 is celebrated as World Hepatitis Day [67]. Celebrate this week with educational seminars, interactive programs with posters, and on-site vaccinations to increase participation.
7) Encouraging peer-to-peer advocacy through student-led nonprofit organizations to create a long-term impact.
8) Provide small gift cards, entry into vaccination lottery, or rewards to promote exciting engagement.
9) Use automated reminders via text, email, or student portal to encourage students to schedule their initial vaccination or missed/upcoming doses.
10) Monitoring and follow-up: Institution should regularly collect vaccination data and maintain tracking system to identify target students requiring initial vaccination or remaining doses.

## 5. Conclusions

The self-reported Hepatitis B vaccination rate in South Asia was much lower than that in developed countries with significant regional variation, with Pakistan leading in a row followed by India, Nepal, and Bangladesh. To improve vaccination status and enhance immune protection against Hepatitis B, major potential areas of intervention include vaccine education and awareness, logistics management, cost-effectiveness, motivation, strict law and policy formulation, and regular monitoring and evaluation.

## Supporting information

S1 Table The 2020 preferred reporting items for systematic reviews and meta-analyses (PRISMA) Checklist. (DOCX)

S2 Table Risk of Bias assessment of included studies using JBI checklist. (DOCX)

S1 Fig Egger’s regression asymmetry test to assess publication bias. (TIF)

S2 Fig Funnel Plot to assess publication bias. (TIF)

S3 Fig Sensitivity analysis by excluding individual study. (TIF)

S1 File Database search strategy. (DOCX)
