# Supplementary material for: Low Hepatitis B vaccination rates among medical students in South Asia: A systematic review and meta-analysis
Source: PLoS One. 2025 Mar 25;20(3):e0320330. doi: 10.1371/journal.pone.0320330 (PMC11936159; doi:10.1371/journal.pone.0320330)
Supplement: S2 Table — (DOCX) [file pone.0320330.s002.docx]

**S2 Table. Risk of Bias assessment of included studies using JBI checklist**

| S. N | JBI questionnaire | Bhattarai et al. 2014 | Shrestha et al. 2020 | Bhattarai et al. 2020 | Dahal et al. 2024 | Sayeed et al. 2007 | Ahmed et al. 2009 | Pantha et al. 2011 | Akhter et al.2016 | Khan et al.2013 | Daud et al.2007 |
| --- | --- | --- | --- | --- | --- | --- | --- | --- | --- | --- | --- |
| 1. | Was the sample frame appropriate to address the target population? | Yes | Yes | Yes | Yes | Yes | Yes | Yes | Yes | Yes | Yes |
| 2. | Were study participants sampled in an appropriate way? | Yes | Yes | Yes | Yes | Unclear | Yes | Yes | No | Unclear | Yes |
| 3 | Was the sample size adequate? | Yes | Yes | Yes | Yes | Yes | No | Yes | Yes | Yes | Yes |
| 4 | Were the study subjects and the setting described in detail? | Yes | Yes | Yes | Yes | No | Yes | Yes | Yes | Yes | Yes |
| 5 | Was the data analysis conducted with sufficient coverage of the identified sample? | Yes | Yes | Yes | Yes | Yes | Yes | Yes | Yes | Yes | Yes |
| 6 | Were valid methods used for the identification of the condition? | Yes | Yes | Yes | Yes | Yes | Yes | Yes | Yes | Yes | Yes |
| 7 | Was the condition measured in a standard, reliable way for all participants? | Yes | Yes | Yes | Yes | Yes | Yes | Yes | Yes | Yes | Yes |
| 8 | Was there appropriate statistical analysis? | Yes | Yes | Yes | Yes | Unclear | Yes | Yes | Yes | Yes | Yes |
| 9 | Was the response rate adequate, and if not, was the low response rate managed appropriately? | Yes | Yes | Yes | Yes | Yes | Yes | Yes | Yes | Yes | Yes |
|  | Overall appraisal | Yes | Yes | Yes | Yes | Yes | Yes | Yes | Yes | Yes | Yes |

| S. No | JBI questionnaire | Diwan et al.2019 | Sahid et al.2019 | Sajid et al.2018 | Khan et al.2018 | Riaz et al.2017 | Malik et al.2017 | Tariq et al.2017 | Butt et al.2015 | Shahbaz et al.2014 | Asif et al. 2009 |
| --- | --- | --- | --- | --- | --- | --- | --- | --- | --- | --- | --- |
| 1. | Was the sample frame appropriate to address the target population? | Yes | Yes | Yes | Yes | Yes | Yes | Yes | Yes | Yes | Yes |
| 2. | Were study participants sampled in an appropriate way? | No | No | No | No | No | No | Yes | Yes | Yes | Yes |
| 3 | Was the sample size adequate? | No | Yes | Yes | Yes | Yes | Yes | Yes | Yes | Yes | Yes |
| 4 | Were the study subjects and the setting described in detail? | No | Yes | Yes | Yes | Yes | Yes | Yes | Yes | Yes | Yes |
| 5 | Was the data analysis conducted with sufficient coverage of the identified sample? | Yes | Yes | Yes | Yes | Yes | Yes | Yes | Yes | Yes | Yes |
| 6 | Were valid methods used for the identification of the condition? | Yes | Yes | Yes | Yes | Yes | Yes | Yes | Yes | Yes | Yes |
| 7 | Was the condition measured in a standard, reliable way for all participants? | Yes | Yes | Yes | Yes | Yes | Yes | Yes | Yes | Yes | Yes |
| 8 | Was there appropriate statistical analysis? | Yes | Yes | Yes | Yes | Yes | Yes | Yes | Yes | Yes | Yes |
| 9 | Was the response rate adequate, and if not, was the low response rate managed appropriately? | Yes | Yes | Yes | Yes | Yes | Yes | Yes | Yes | Yes | Yes |
|  | Overall appraisal | Yes | Yes | Yes | Yes | Yes | Yes | Yes | Yes | Yes | Yes |

| S. No | JBI questionnaire | Khurram et al.2008 | Nasir et al.2000 | Sachidananda et al.2018 | Srinivas et al.2021 | Pratap et al.2024 | Gautam et al.2022 | Agarwal et al.2018 | Pulluri et al.2017 | Gupta et al.2017 | Avachat et al.2017 |
| --- | --- | --- | --- | --- | --- | --- | --- | --- | --- | --- | --- |
| 1. | Was the sample frame appropriate to address the target population? | Yes | Yes | Yes | Yes | Yes | Yes | Yes | Yes | Yes | Yes |
| 2. | Were study participants sampled in an appropriate way? | Yes | Yes | Yes | Yes | Unclear | Yes | Yes | Unclear | Unclear | Yes |
| 3 | Was the sample size adequate? | Yes | Yes | Yes | Yes | Unclear | Yes | Yes | Yes | Yes | Yes |
| 4 | Were the study subjects and the setting described in detail? | Yes | Yes | Yes | Yes | Yes | Yes | Yes | Yes | Yes | Yes |
| 5 | Was the data analysis conducted with sufficient coverage of the identified sample? | Yes | Yes | Yes | Yes | Yes | Yes | Yes | Yes | Yes | Yes |
| 6 | Were valid methods used for the identification of the condition? | Yes | Yes | Yes | Yes | Yes | Yes | Yes | Yes | Yes | Yes |
| 7 | Was the condition measured in a standard, reliable way for all participants? | Yes | Yes | Yes | Yes | Yes | Yes | Yes | Yes | Yes | Yes |
| 8 | Was there appropriate statistical analysis? | Yes | Yes | Yes | Yes | Yes | Yes | Yes | Yes | Yes | Yes |
| 9 | Was the response rate adequate, and if not, was the low response rate managed appropriately? | Yes | Yes | Yes | Yes | Yes | Yes | Yes | Yes | Yes | Yes |
|  | Overall appraisal | Yes | Yes | Yes | Yes | Yes | Yes | Yes | Yes | Yes | Yes |

| S. No | JBI questionnaire | Acchammachary et al.2017 | Batra et al.2015 | Pattanshetty et al.2010 | Thote et al.2023 | Rathi et al.2018 | Wadekar et al.2019 | Nowreen at al.2019 | Mishra et al.2019 | Dawar et al.2019 | Chhabra et al.2019 |
| --- | --- | --- | --- | --- | --- | --- | --- | --- | --- | --- | --- |
| 1. | Was the sample frame appropriate to address the target population? | Yes | Yes | Yes | Yes | Yes | Yes | Yes | Yes | Yes | Yes |
| 2. | Were study participants sampled in an appropriate way? | Yes | Unclear | Unclear | Yes | No | No | No | Yes | Yes | Yes |
| 3 | Was the sample size adequate? | Yes | Yes | Yes | Yes | Yes | Yes | Yes | Yes | Yes | Yes |
| 4 | Were the study subjects and the setting described in detail? | Yes | Yes | No | Yes | Yes | Yes | Yes | Yes | Yes | Yes |
| 5 | Was the data analysis conducted with sufficient coverage of the identified sample? | Yes | Yes | Yes | Yes | Yes | Yes | Yes | Yes | Yes | Yes |
| 6 | Were valid methods used for the identification of the condition? | Yes | Yes | Yes | Yes | Yes | Yes | Yes | Yes | Yes | Yes |
| 7 | Was the condition measured in a standard, reliable way for all participants? | Yes | Yes | Yes | Yes | Yes | Yes | Yes | Yes | Yes | Yes |
| 8 | Was there appropriate statistical analysis? | Yes | Yes | Yes | Yes | Yes | Yes | Yes | Yes | Yes | Yes |
| 9 | Was the response rate adequate, and if not, was the low response rate managed appropriately? | Yes | Yes | Unclear | Yes | Yes | Yes | Yes | Yes | Yes | Yes |
|  | Overall appraisal | Yes | Yes | Yes | Yes | Yes | Yes | Yes | Yes | Yes | Yes |

| S. No | JBI questionnaire | Jayakiruthiga et al.2018 | Giri et al.2016 | Hussain et al.2016 | Pavani et al.2015 | Paul et al.2015 | Biradar et al.2015 | Baig et al.2015 | Suganthi et al.2014 | Gujjarlapudi et al.2013 | Singh et al.2011 |
| --- | --- | --- | --- | --- | --- | --- | --- | --- | --- | --- | --- |
| 1. | Was the sample frame appropriate to address the target population? | Yes | Yes | Yes | Yes | Yes | Yes | Yes | Yes | Yes | Yes |
| 2. | Were study participants sampled in an appropriate way? | Yes | Yes | Unclear | No | Yes | Yes | No | Unclear | No | Yes |
| 3 | Was the sample size adequate? | Yes | Yes | Yes | Yes | Yes | Yes | Yes | Yes | Yes | Yes |
| 4 | Were the study subjects and the setting described in detail? | Yes | Yes | Yes | Yes | Yes | Yes | Yes | Yes | Yes | Yes |
| 5 | Was the data analysis conducted with sufficient coverage of the identified sample? | Yes | Yes | Yes | Yes | Yes | Yes | Yes | Yes | Yes | Yes |
| 6 | Were valid methods used for the identification of the condition? | Yes | Yes | Yes | Yes | Yes | Yes | Yes | Yes | Yes | Yes |
| 7 | Was the condition measured in a standard, reliable way for all participants? | Yes | Yes | Yes | Yes | Yes | Yes | Yes | Yes | Yes | Yes |
| 8 | Was there appropriate statistical analysis? | Yes | Yes | Yes | Yes | Yes | Yes | Yes | Yes | Yes | Yes |
| 9 | Was the response rate adequate, and if not, was the low response rate managed appropriately? | Yes | Yes | Yes | Yes | Yes | Yes | Yes | Yes | Yes | Yes |
|  | Overall appraisal | Yes | Yes | Yes | Yes | Yes | Yes | Yes | Yes | Yes | Yes |
