# Supplementary figures and images for: Low Hepatitis B vaccination rates among medical students in South Asia: A systematic review and meta-analysis
Source: PLoS One. 2025 Mar 25;20(3):e0320330. doi: 10.1371/journal.pone.0320330 (PMC11936159; doi:10.1371/journal.pone.0320330)

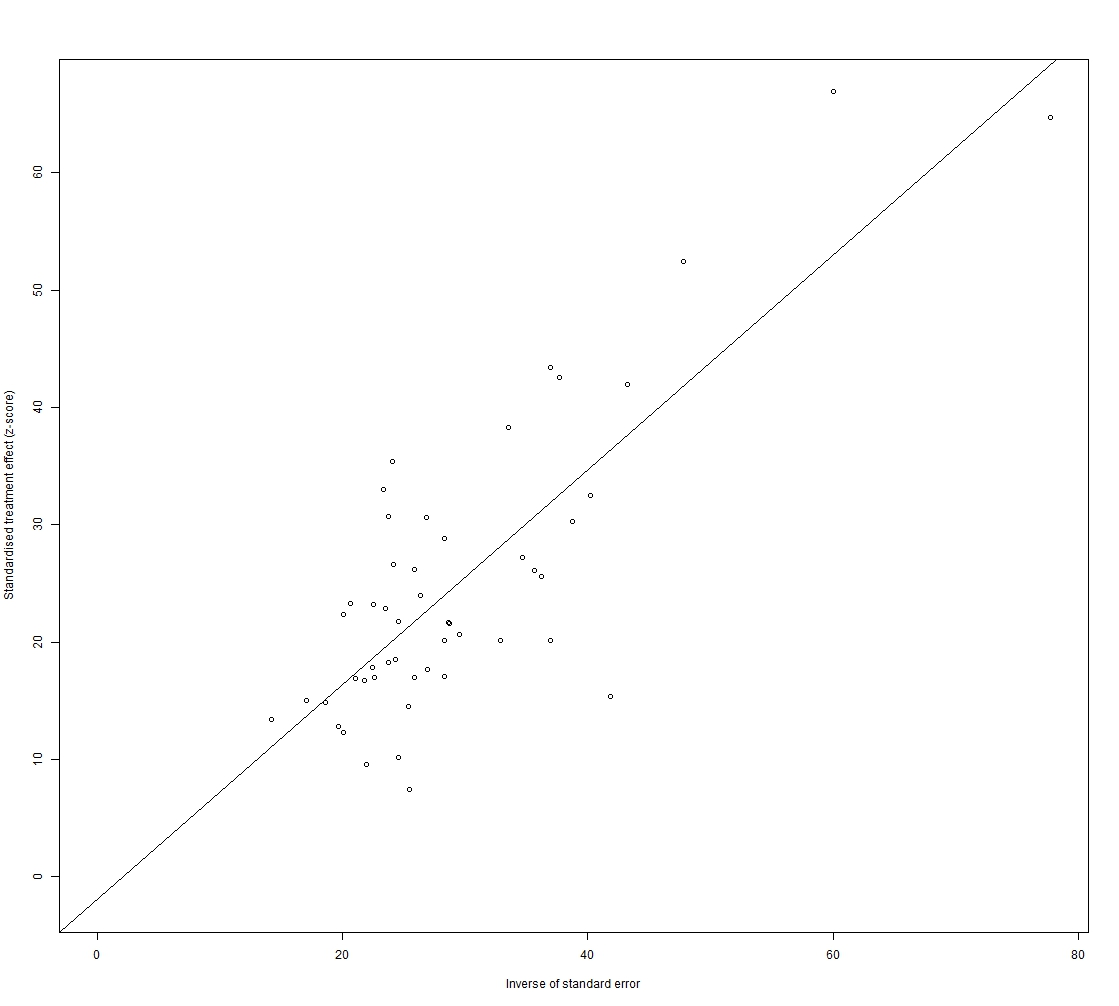

Supplement: S1 Fig — (TIF) [file pone.0320330.s003.tif]

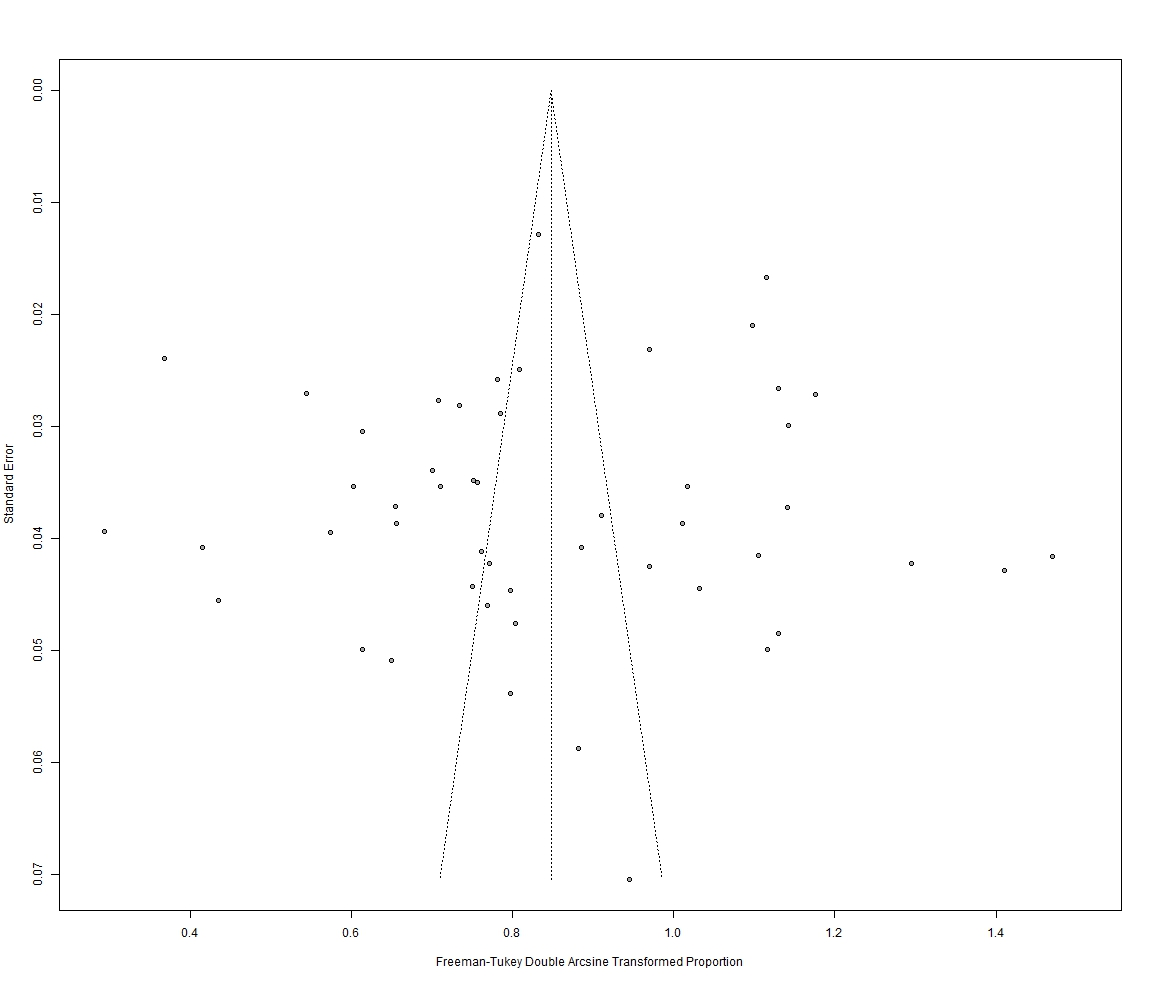

Supplement: S2 Fig — (TIF) [file pone.0320330.s004.tif]

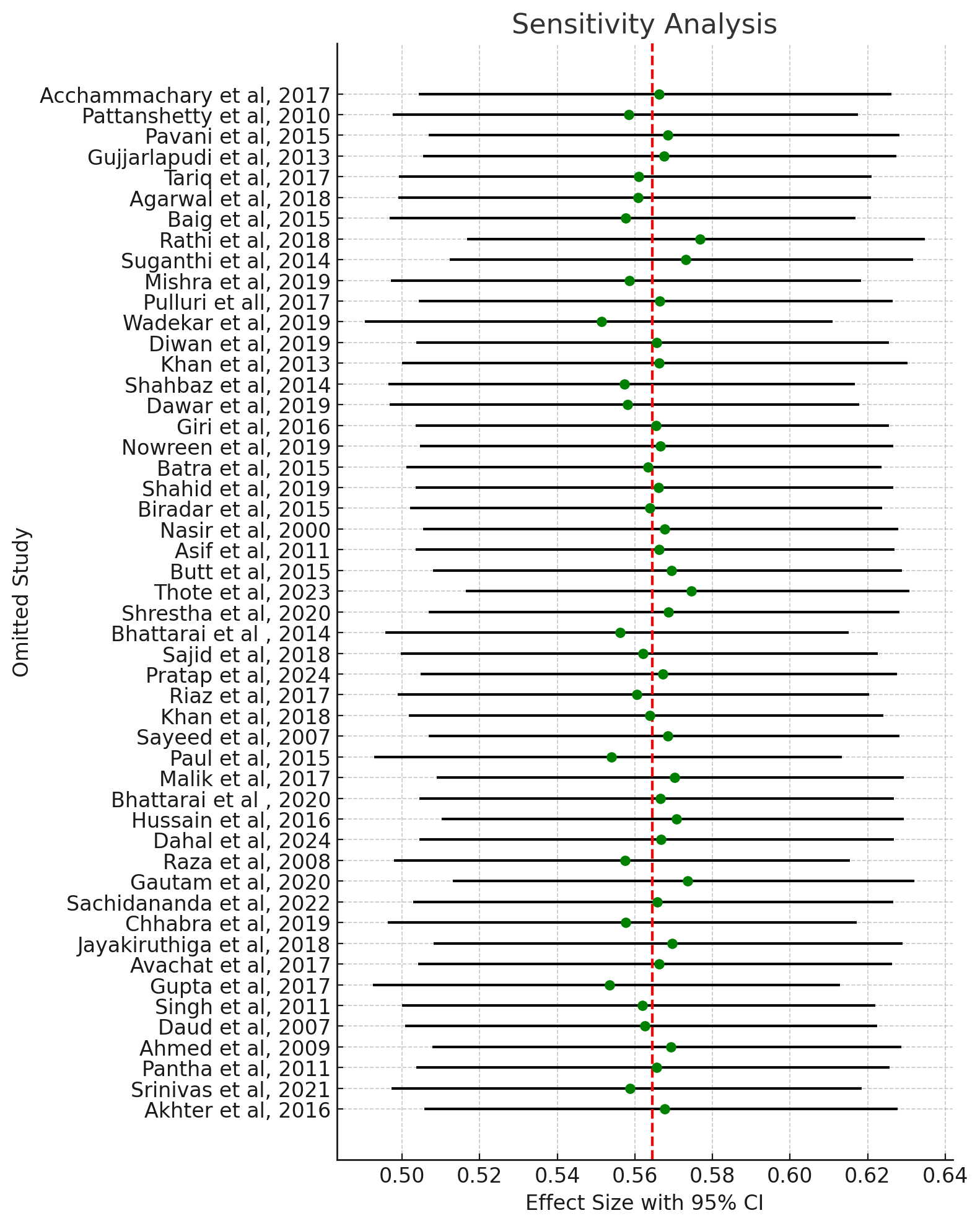

Supplement: S3 Fig — (TIF) [file pone.0320330.s005.tif]
