# Supplementary material for: Low Hepatitis B vaccination rates among medical students in South Asia: A systematic review and meta-analysis
Source: PLoS One. 2025 Mar 25;20(3):e0320330. doi: 10.1371/journal.pone.0320330 (PMC11936159; doi:10.1371/journal.pone.0320330)
Supplement: S1 File — (DOCX) [file pone.0320330.s006.docx]

**S1 File. Database search strategy:**

| **Databases** | **Search Strategy** | **Number of studies** |
| --- | --- | --- |
| PubMed | Inception to July 15, 2024  (("Hepatitis B Vaccines"[Mesh] OR "Hepatitis B" OR HBV ) AND (Vaccination[Mesh] OR Immunization OR "Vaccination Coverage" ) AND ("Students, Medical"[Mesh] OR "Medical Students" OR "Healthcare Students" ) AND (Nepal OR India OR Bangladesh OR Pakistan OR "Sri Lanka" OR Bhutan OR Maldives OR Afghanistan )) | 23 |
| PubMed Central | Inception to July 15, 2024  (("Hepatitis B Vaccines"[Mesh] OR "Hepatitis B" OR HBV ) AND (Vaccination[Mesh] OR Immunization OR "Vaccination Coverage" ) AND ("Students, Medical"[Mesh] OR "Medical Students" OR "Healthcare Students" ) AND (Nepal OR India OR Bangladesh OR Pakistan OR "Sri Lanka" OR Bhutan OR Maldives OR Afghanistan )) y | 614 |
| Scopus | Inception to July 15, 2024  ((INDEXTERMS("Hepatitis B Vaccines") OR "Hepatitis B" OR HBV ) AND (INDEXTERMS(Vaccination) OR Immunization OR "Vaccination Coverage" ) AND (INDEXTERMS("Students, Medical") OR "Medical Students" OR "Healthcare Students" ) AND (Nepal OR India OR Bangladesh OR Pakistan OR "Sri Lanka" OR Bhutan OR Maldives OR Afghanistan )) | 514 |
| Embase (Ovid) | 1974 to 2024 July 15  1 ('hepatitis b vaccine' or 'hepatitis b' or HBV).mp. [mp=title, abstract, heading word, drug trade name, original title, device manufacturer, drug manufacturer, device trade name, keyword heading word, floating subheading word, candidate term word] 206439  2 ('hepatitis b vaccine' or 'hepatitis b' or HBV).mp. [mp=title, abstract, heading word, drug trade name, original title, device manufacturer, drug manufacturer, device trade name, keyword heading word, floating subheading word, candidate term word] 206439  3 ('vaccination' or 'immunization' or 'vaccination coverage').mp. [mp=title, abstract, heading word, drug trade name, original title, device manufacturer, drug manufacturer, device trade name, keyword heading word, floating subheading word, candidate term word] 475065  4 ('medical student' or 'health care student').mp. [mp=title, abstract, heading word, drug trade name, original title, device manufacturer, drug manufacturer, device trade name, keyword heading word, floating subheading word, candidate term word] 100818  5 (Nepal or India or Bangladesh or Pakistan or 'Sri Lanka' or Bhutan or Maldives or Afghanistan).mp. [mp=title, abstract, heading word, drug trade name, original title, device manufacturer, drug manufacturer, device trade name, keyword heading word, floating subheading word, candidate term word] 400320  6 2 and 3 and 4 and 5 25 | 25 |
| **Medline (Ovid MEDLINE® Epub Ahead of Print, In-Process & Other Non-Indexed Citations, Ovid MEDLINE® Daily and Ovid MEDLINE®)** | Inception to July 15, 2024  ((exp "Hepatitis B Vaccines"/ OR "Hepatitis B" OR HBV ) AND (exp Vaccination/ OR Immunization OR "Vaccination Coverage" ) AND (exp "Students, Medical"/ OR "Medical Students" OR "Healthcare Students" ) AND (Nepal OR India OR Bangladesh OR Pakistan OR "Sri Lanka" OR Bhutan OR Maldives OR Afghanistan )) | 10 |
| **Cinhl** | Inception to July 15, 2024  (((MH "Hepatitis B Vaccines+") OR "Hepatitis B" OR HBV ) AND ((MH Vaccination+) OR Immunization OR "Vaccination Coverage" ) AND ((MH "Students, Medical+") OR "Medical Students" OR "Healthcare Students" ) AND (Nepal OR India OR Bangladesh OR Pakistan OR "Sri Lanka" OR Bhutan OR Maldives OR Afghanistan )) | 2 |
| **Google Scholar** | Inception to July 15, 2024  (((MH "Hepatitis B Vaccines+") OR "Hepatitis B" OR HBV ) AND ((MH Vaccination+) OR Immunization OR "Vaccination Coverage" ) AND ((MH "Students, Medical+") OR "Medical Students" OR "Healthcare Students" ) AND (Nepal OR India OR Bangladesh OR Pakistan OR "Sri Lanka" OR Bhutan OR Maldives OR Afghanistan )) | 827 |
